# Robotic-assisted surgical removal of retroperitoneal schwannoma by transmesocolic access

**DOI:** 10.1590/S1677-5538.IBJU.2018.0624

**Published:** 2020-01-13

**Authors:** Marcos Tobias-Machado, William Enrique Pertuz Genes, Cristiano Linck Pazeto, Eliney Ferreira Faria, Hamilton Campos Zampolli

**Affiliations:** 1 Faculdade de Medicina do ABC, Santo André, SP, Brasil; 2 Departamento de Urologia, Faculdade de Medicina do ABC, Santo André, SP, Brasil; 3 Departamento de Urologia, Hospital de Câncer de Barretos, Barretos, SP, Brasil; 4 Divisão de Urologia, Instituto do Câncer Arnaldo Veira de Carvalho, São Paulo, SP, Brasil

## Abstract

**Introduction:**

Schwannoma are usually benign tumors, most of the cases are asymptomatic, and others may present symptoms by compression. In the literature robotic surgery were described in 8 cases. We emphasize that robotic surgery improves visualization and enable the performance of this procedure.

**Objectives:**

Describe and evaluate the results and benefits of resection of a retroperitoneal tumor by means of robotic surgery by transmesocolic access.

**Materials and methods:**

We present a case of a 34 year old patient, with low back pain, who were diagnosed with a retroperitoneal tumor in which an incisional biopsy by laparoscopy was previously performed with the diagnosis of schwannoma, measuring 4.1cm x 3 cm next to the left renal hilum and near to abdominal aorta. Robotic surgery was performed. It was possible to localize the vena cava, aorta and left renal hilum and consequently it was possible to preserve the adjacent structures. The resection of the tumor was carried out carefully allowing complete tumor resection.

**Results:**

The total of procedure time was 230 minutes, blood loss was 60ml, 1 day of hospital stay without complications. The histopathological findings confirmed benign Schwannoma.

**Conclusion:**

The maximization of robotic surgery images offers dexterity and dissection capacity, required for the complex dissection of masses in the retroperitoneum. It is safe and effective for removing benign retroperitoneal schwannomas when performed by experienced surgeons. This transmesocolic robotic assisted surgical approach could be an option in selected cases.

## ARTICLE INFO

**Available at: http://www.intbrazjurol.com.br/video-section/20180624_Tobias-Machado_et_al**

**Int Braz J Urol. 2020; 46 (Video #5): 143-144**

